# Mutation of a Conserved Nuclear Export Sequence in Chikungunya Virus Capsid Protein Disrupts Host Cell Nuclear Import

**DOI:** 10.3390/v9100306

**Published:** 2017-10-20

**Authors:** Susan C. Jacobs, Adam Taylor, Lara J. Herrero, Suresh Mahalingam, John K. Fazakerley

**Affiliations:** 1 The Roslin Institute, The University of Edinburgh, Midlothian EH25 9RG, UK; jacobs_susan@hotmail.com; 2 Institute for Glycomics, Gold Coast Campus, Griffith University, Nathan, QLD 4212, Australia; a.taylor1@griffith.edu.au (A.T.); l.herrero@griffith.edu.au (L.J.H.)

**Keywords:** alphavirus, chikungunya virus, capsid protein, nuclear export

## Abstract

Transmitted by mosquitoes; chikungunya virus (CHIKV) is responsible for frequent outbreaks of arthritic disease in humans. CHIKV is an arthritogenic alphavirus of the *Togaviridae* family. Capsid protein, a structural protein encoded by the CHIKV RNA genome, is able to translocate to the host cell nucleus. In encephalitic alphaviruses nuclear translocation induces host cell shut off; however, the role of capsid protein nuclear localisation in arthritogenic alphaviruses remains unclear. Using replicon systems, we investigated a nuclear export sequence (NES) in the N-terminal region of capsid protein; analogous to that found in encephalitic alphavirus capsid but uncharacterised in CHIKV. The chromosomal maintenance 1 (CRM1) export adaptor protein mediated CHIKV capsid protein export from the nucleus and a region within the N-terminal part of CHIKV capsid protein was required for active nuclear targeting. In contrast to encephalitic alphaviruses, CHIKV capsid protein did not inhibit host nuclear import; however, mutating the NES of capsid protein (∆NES) blocked host protein access to the nucleus. Interactions between capsid protein and the nucleus warrant further investigation.

## 1. Introduction

Chikungunya virus (CHIKV) is a mosquito borne arthritogenic alphavirus responsible for numerous outbreaks of arthritic disease with significant human morbidity. Widely regarded as a nonfatal self-limiting disease, chikungunya gained global attention during the 2005–2006 Indian Ocean outbreak due to the severe and atypical nature of clinical symptoms [1,2]. Since then, CHIKV has spread rapidly in a relatively short period of time, most recently making incursions into the Americas where local transmission has been identified in over 40 countries or territories [3].

Belonging to the *Togaviridae* family, CHIKV is an arthritogenic alphavirus. Other arthritogenic alphaviruses include Sindbis virus (SINV), o’nyong nyong virus (ONNV), and Ross River virus. Encephalitic alphaviruses—such as Eastern, Western, and Venezuelan equine encephalitis viruses—are responsible for sporadic cases of human and equine neurological disease and are largely found in the western hemisphere. CHIKV, as with all alphaviruses, has a single strand positive-sense ~12-kb RNA genome. Genomic RNA serves as the mRNA for translation of four non-structural proteins (nsP1–4), which form the replicative enzyme complex responsible for viral genome replication, and transcription of a subgenomic RNA. The latter encodes five virus structural proteins (capsid protein, E3, E2, 6K/TF, and E1). The non-structural and structural proteins are each translated as polyprotein precursors that undergo proteolytic cleavage to form the mature viral proteins. The multifunctional capsid protein has a number of key roles. Through the activity of a serine protease catalytic site, the capsid protein cleaves itself from the nascent structural polyprotein. In a structural capacity, capsid protein specifically recognizes the packaging signals present in viral genomic RNA, allowing assembly of the nucleocapsid core [4]. There are though functional differences between arthritogenic and encephalitic alphavirus capsid proteins. In mammalian cells, encephalitic alphavirus capsid proteins inhibit cellular transcription, while for arthritogenic viruses cellular transcription is antagonized by nsP2 [5,6]. The capsid protein of encephalitic alphaviruses is highly cytotoxic and this protein’s ability to shutdown host transcription is closely linked to an interaction with the nuclear pore complex [5,7].

Both arthritogenic and encephalitic alphavirus capsid proteins traffic to the mammalian host cell nucleus [5,8]. Disruption of capsid protein nuclear trafficking in encephalitic alphaviruses can impact on virulence and pathogenesis in vivo [5]. Despite this, little is known of this importance of capsid protein nuclear trafficking in arthritogenic alphaviruses including CHIKV.

The presence of nuclear import and export signals in the capsid proteins of a number of alphaviruses, including CHIKV, have been documented [7,8,9,10,11]. However, the regions within capsid protein responsible for nuclear export have been found to vary greatly between different alphaviral clades and species. Here, using site directed mutagenesis, we identify amino acid residues required for nuclear export and, using replicon systems, we show that mutating the nuclear export sequence of CHIKV capsid protein blocks host protein access to the nucleus.

## 2. Materials and Methods

### 2.1. Oligonucleotides, Plasmids, and Antibodies

Insertion of a PCR amplicon encoding the CHIKV capsid gene, from primers CHIKV-CAP-PCR XhoI F (GGCCCTCGAGAGTTCATCCCAACC) and CHIKV-CAP-PCR HindIII R (GCGCAAGCTTTACCACTCTTCGGCCC), into the XhoI-HindII sites in pEGFP-C1 generated pEGFP-CHIKV capsid. The mutations L51A and M53A within the CHIKV capsid protein encoding region were generated using overlapping PCR products. The primers CHIK-CAP-PCR XhoI F and ∆NES-L51A, M53A R (CGCGCgcTGTCgcTTTATTAACTGCTGAGATCAG) were used to generate the 5′ region of capsid containing the mutations at its 3′. While, the primers ∆NES-L51A, M53A F (AAAgcGACAgcGCGCGCGGTACCACAACAG), and CHIK-CAP-PCR HindIII R were used to generate the 3′ region of capsid encoding the mutations at its 5′. Following gel purification these PCR products were mixed and included in a PCR reaction with the primers CHIK-CAP-PCR XhoI F and CHIK-CAP-PCR HindIII R. The resultant amplicon was cloned into pEGFP-C1 between the XhoI and HindIII site to generate pEGFP-CHIKV capsid-∆NES.

The plasmid pSP6-ICRES1-ΔNES was generated by subcloning the AgeI-SfiI fragment containing the subgenomic promoter (SGP) and 5′ of the capsid gene from pSP6-ICRES1 (generated from the CHIKV strain LR2006_OPY1 and kindly provided by Andres Merits at the University of Tartu) into pCDNA.31(+) (Invitrogen, Carlsbad, CA, USA) to generate the pCDNA3.1(+)-SGP-CAP shuttle vector. The nucleic acid fragment encoding the capsid nuclear export sequence (NES) contained on a KpnI fragment was exchanged with that from the plasmid pEGFP-CHIKV capsid-ΔNES, described above. The AgeI-SflI fragment containing the ΔNES was returned to pSP6-ICRES1 to generate pSP6-ICRES1-ΔNES.

All CHIKV replicons encoding the capsid protein were constructed using pSP6-ChikRepl-SG2, kindly provided by Andres Merits at the University of Tartu. A NheI-SmaI fragment encoding the EGFP-capsid fusion (WT and ΔNES mutant) was removed from pEGFP-CHIKV-Capsid/pEGFP-CHIKV-Capsid ΔNES and cloned into pSP6-ChikRepl-SG2 using the unique AvrII and PmeI sites. This placed CHIKV capsid under the control of the subgenomic promoter of the replicon vector.

The CHIKV replicon encoding fusion protein consisting of four copies of mCherry protein harbouring the SV40 NLS was generated in three stages. Firstly, a PCR amplicon encoding mCherry was cloned into pSP6-CHKVRep-SG-ZsGreen to replace the ZsGreen gene using the AvrII and PmeI sites. Secondly, oligonucleotides encoding three copies of the SV40 NLS were synthesized and cloned at the 5′ of the mCherry gene using the SgrAI site located at the 3′ of mCherry and PmeI of the plasmid to generate SGP-AvrII-mCherry-SgrAI-3X NLS-PmeI. A control replicon lacking the 3XNLS was also generated.

All Venezuelan equine encephalitis virus (VEEV) capsid constructs were prepared as the CHKV EGFP-Capsid constructs. The VEEV capsid gene was synthesized by Dundee Cells Products (Dundee) based on the Trinidad Donkey/TC83 (GI:323714/GI:323714) vaccine strain and used as a template for all VEEV PCR reactions. The sequences of these and other constructs were verified by sequencing. Antibodies against capsid proteins were kindly provided by Roy Hall at The University of Queensland. Antibodies against nsP2 were kindly provided by Andres Merits at the University of Tartu.

### 2.2. Cell Culture and Virus Propagation

BHK-21 cells (Sigma-Aldrich, St. Louis, MO, USA) were propagated in Opti-MEM (Life Technologies, Carlsbad, CA, USA) supplemented with 3% Fetal bovine serum (FBS) (Bovogen, Melbourne, Australia). Infectious viruses were rescued by linearization of icDNA containing plasmids with NotI and in vitro transcription from the SP6 promoter using the AmpliCapTM SP6 High Yield Message Maker Kit (CELLSRIPT, Madison, WI, USA). Transcripts were electroporated into Vero cells and supernatants collected and titrated by plaque assay as described previously [12]. Plasmid transfections were carried out with Lipofectamine^®^ 2000 (Life Technologies), according to the manufacturer’s protocol.

### 2.3. Immunofluorescence Microscopy

Cells were grown on polylysine-treated coverslips. For Leptomycin B treatment, 45 nM of Leptomycin B was added to cells for 30 min at 37 °C immediately prior to imaging. Cells were fixed in 4% paraformaldehyde and permeabilised in 1% Triton X-100. Cells were then blocked in 1% bovine serum albumin (BSA) made in PBS and incubated at 37 °C for 1 h. Primary antibodies were diluted 1:100 in 1% BSA and incubated with the cells for 1 h at 37 °C. Alexa Fluor 488 secondary antibody (Invitrogen) was diluted 1:500 in 1% BSA and incubated with the cells for 1 h at 37 °C. Coverslips were mounted in Vectorshield mounting medium (Vector Laboratories, Burlingame, CA, USA) and staining was visualized on an Olympus FluoView™ FV1000 confocal microscope.

## 3. Results

### 3.1. Localisation of CHIKV Capsid Protein to the Nucleus and Identification of the Minimal Nuclear Export Sequence

**Figure 1:**
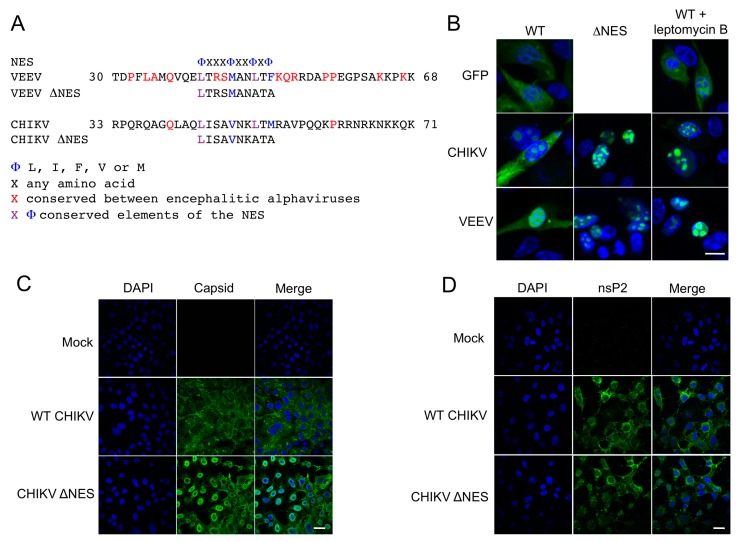
Identification of the CHIKV capsid protein minimal nuclear export sequence. (**A**) Amino acid sequence alignment of CHIKV capsid putative nuclear export sequence against VEEV capsid; (**B**) BHK-21 cells were transfected with pEGFP, pEGFP-CHIKV capsid, pEGFP-VEEV capsid (in the presence or absence of Leptomycin B), pEGFP-CHIKV capsid-∆NES, or pEGFP-VEEV capsid-∆NES. After 24 h, cells were treated with Leptomycin B for 30 min at 37 °C prior to fixing, and EGFP fluorescence was analyzed by direct visualization. The white bar represents 10 µm; (**C**,**D**) BHK-21 cells were infected with WT CHIKV or CHIKV ΔNES. After 24 h cells were fixed, permeabilized, and capsid protein (**C**) and nsP2 (**D**) localization were analysed by indirect immunofluorescence using anti-CHIKV capsid and anti-nsP2 antibodies. Images are representative of three independent experiments and at least six fields of view. The white bar represents 25 µm.

Amino acid sequence alignment of CHIKV capsid with that of the encephalitic alphavirus VEEV (Venezuelan equine encephalitis virus) [7] identified a putative nuclear export sequence (NES) in the N-terminal part of the protein (Figure 1A). To investigate whether this motif was responsible for CHIKV capsid protein nuclear export, site-directed mutagenesis was performed altering the wild type (WT) residues L51 and M53 to alanines. Mutations were performed in recombinant pEGFP-capsid to allow WT and mutants protein localization to be visualized directly via GFP fluorescence. WT CHIKV EGFP-capsid fusion protein was distributed between the cytoplasm and nucleus (Figure 1B). Mutation of the putative NES (∆NES) resulted in the EGFP-∆NES capsid fusion protein signal being solely nuclear (Figure 1B). Results with CHIKV capsid protein were indistinguishable from those obtained with similar VEEV capsid constructs replicated from previous studies [7] and shown in Figure 1B. Furthermore, both WT CHIKV capsid and WT VEEV capsid accumulated in the nucleus in cells treated with the nuclear export inhibitor leptomycin B (Figure 1B). Leptomycin B disrupts nuclear export mediator protein CRM1 binding to capsid protein, suggesting that both VEEV and CHIKV capsid protein are actively exported from the nucleus.

To examine the localization of CHIKV capsid protein and nsP2 in virus infected cells, CHIKV ∆NES virus was engineered and BHK-21 cells infected with this and as a comparison WT CHIKV. No obvious difference was observed between the replication kinetics of CHIKV WT and CHIKV ΔNES in BHK-21 cells. At 24 h post infection, cells were fixed and permeabilized, and protein localization determined by indirect immunofluorescence using anti-CHIKV capsid and nsP2 antibodies. Capsid protein did not accumulate in the nucleus of WT CHIKV infected cells (Figure 1C). In contrast, significant quantities of capsid protein were, detected in the nuclei of cells infected with CHIKV ΔNES (Figure 1C), with capsid protein accumulating at the nuclear periphery. The localization of nsP2, a viral protein also shown to transit the nucleus during infection, was mostly cytoplasmic and not altered by mutation of the capsid protein NES (Figure 1D). These results indicate that WT CHIKV capsid protein is actively and rapidly exported from the cell nucleus during infection.

### 3.2. A Functional CHIKV Capsid Protein Nuclear Export Sequence Is Required for Nuclear Import of Cellular Proteins

**Figure 2:**
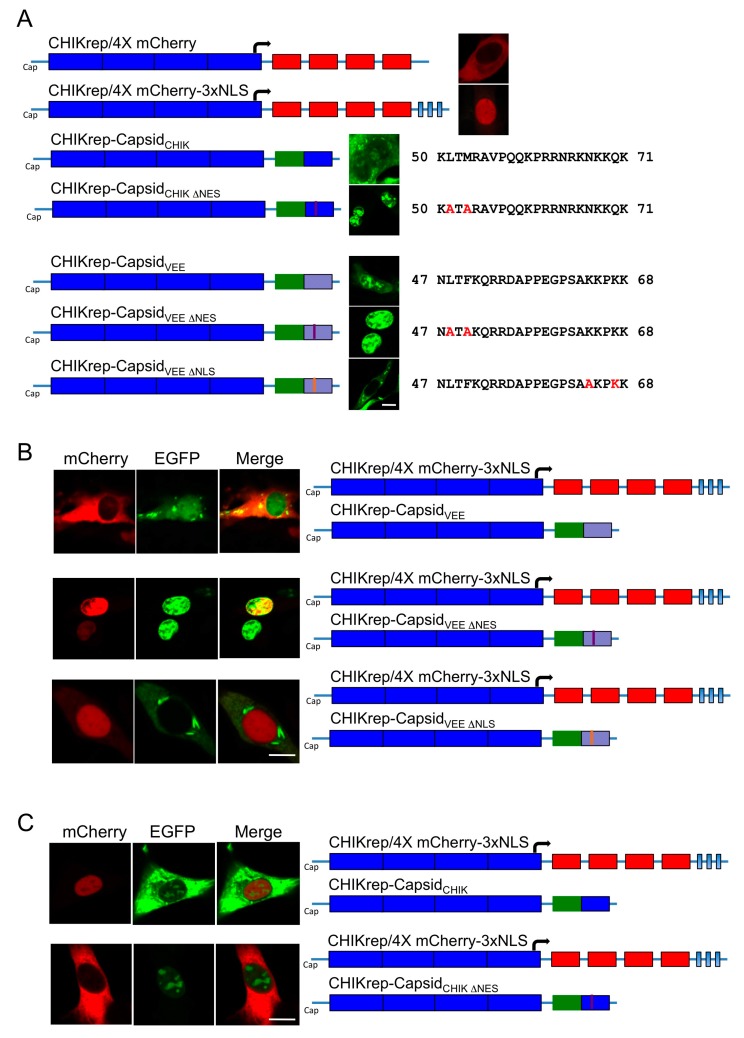
Functional CHIKV capsid protein NES is required for nuclear import. (**A**) BHK-21 cells were electroporated with CHIKV replicons (solid blue) encoding four mCherry proteins (CHIKrep/4X mCherry) (solid red) or four mCherry proteins plus three copies of the SV40 nuclear localization signal (CHIKrep/4X mCherry-3xNLS) (solid light blue) under control of subgenomic promoter, together with either; (**B**) EGFP-VEEV capsid (hatched blue), EGFP-VEEV ΔNES capsid (mauve bar) or EGFP-VEEV ΔNLS capsid (orange bar), or (**C**) EGFP-CHIKV capsid, EGFP-CHIKV ΔNES capsid. Cells were fixed after 24 h and EGFP and mCherry fluorescence analyzed by direct visualization. Images are representative of three independent experiments and at least six fields of view. The white bars represent 15 µm.

Using a VEEV replicon system, previous studies have shown that VEEV capsid protein is able of blocking transport across the nuclear pore when complexed with CRM1 and importin α/β [7]. To determine whether CHIKV capsid has the same activity, a series of constructs to report on nuclear access and to compare the activity of the CHIKV and VEEV capsid proteins were constructed, these are shown in Figure 2A. As a reporter of nuclear access, we used CHIKV replicons encoding four copies of mCherry protein (CHIKrep/4X mCherry) or four mCherry proteins plus three copies of the SV40 nuclear localization signal (CHIKrep/4X mCherry-3xNLS) under the control of a subgenomic promoter (Figure 2A). The reporter 4XmCherry fusion protein localized to the nucleus only when actively transported by the 3xNLS (Figure 2A). As with the studies reported earlier, replicons expressing the CHIKV capsid showed both nuclear and cytoplasmic location of capsid and this was converted to exclusively nuclear in the ΔNES replicon. In the case of CHIKV replicons expressing VEEV capsid protein, capsid was again both nuclear and cytoplasmic and was converted to exclusively nuclear, or exclusively cytoplasmic, by mutation of the NES or of the NLS, respectively (Figure 2A).

In co-transfection experiments, where reporter and capsid encoding replicons were found in the same cell, CHIKV replicon encoding EGFP tagged-VEEV capsid protein (EGFP-VEEV-Cap) was able to block active transport of 4X mCherry-3xNLS into the nucleus, indicating that the origin of the nsP2 protein is not important in the ability of VEEV capsid to block nuclear transport (Figure 2B). In the CHIKV background, EGFP-VEEV-capsid lacking a nuclear export sequence or nuclear localization signal, as described by Atasheva et al., was unable to block entry of 4X mCherry-3xNLS into the nucleus demonstrating that, as with the VEEV replicon, the VEEV capsid-CRM1-Importin α/β to the blockage reported here (Figure 2B).

In contrast to the EGFP-VEEV-capsid, EGFP-CHIKV capsid did not block active transport of 4X mCherry-3xNLS to the nucleus (Figure 2C). When the NES was deleted from EGFP-CHIKV-Cap, CHIKV capsid was detected in the nucleus suggesting that the interior of the nucleus remains accessible. Unexpectedly however, CHIKV-∆NES-capsid did block transport of 4X mCherry-3xNLS to the nucleus (Figure 2B).

### 3.3. The N-Terminus Is Required for Active Nuclear Targeting of CHIKV Capsid Protein

In the above studies, EGFP fused to the N-terminus of CHIKV capsid protein was used to demonstrate active transport of CHIKV capsid to the nucleus and capsid protein was present in both the cytoplasm and nucleus of BHK-21 cells (Figure 2A,C). The same was observed in HeLa cells. In the nucleus, capsid protein accumulated in foci reminiscent of the nucleolus. This was particularly apparent with capsid protein lacking a nuclear export sequence (∆NES) (Figure 2C). To further investigate the subcellular localization of capsid protein, truncated mutants (T1, T2, T3, and T4) lacking portions of the N or C terminus of capsid protein were constructed and their subcellular localization analyzed.

**Figure 3:**
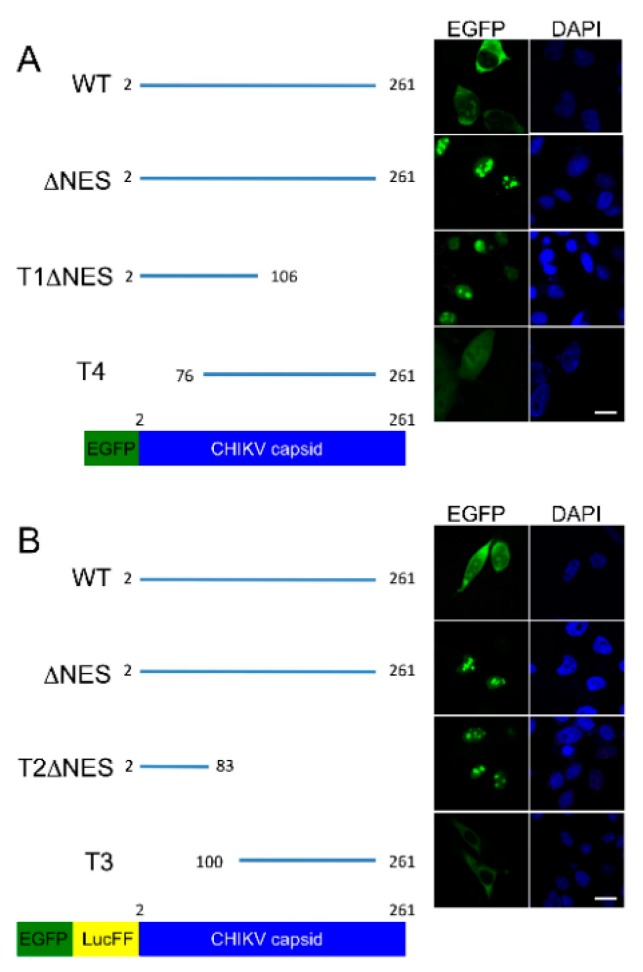
The N-terminus is required for active nuclear targeting of CHIKV capsid protein. (**A**) BHK-21 cells were transfected with either CHIKV WT EGFP-capsid, EGFP-∆NES, EGFP-T1∆NES, EGFP-T4 or, to ensure nuclear translocation was not due to passive diffusion; (**B**) CHIKV WT EGFP-LucFF-capsid, EGFP-LucFF-∆NES, EGFP-LucFF-T2∆NES, EGFP-LucFF-T3, which contain an insertion of the luciferase firefly gene (LusFF) between EGFP and the capsid gene to increase the overall molecular weight of the recombinant protein. After 24 h, cells were fixed and EGFP and fluorescence analyzed by direct visualization. Images are representative of three independent experiments and at least six fields of view. The white bars represent 15 µm.

Localization of the N-terminal part (aa residues 2–106) of CHIKV capsid protein encoded by T1 that also contains a mutated NES (T1∆NES; NES was removed to demonstrate capsid protein nuclear localization more clearly) was similar to that of full-length capsid with ∆NES, although weak cytoplasmic staining was observed in a small proportion of cells (Figure 3A). Truncation T4 encodes the C-terminus of CHIKV capsid protein from A76 residue; corresponding protein located to both the nucleus and cytoplasm (Figure 3A). These results suggest that, like WT capsid protein, truncated proteins T1∆NES and T4 may be actively transported to the nucleus and that a nuclear localization signal may reside between residues A76 and C106.

To ensure nuclear localization was not due to passive diffusion constructs that contain an in-frame insertion of the sequence encoding for firefly luciferase (LucFF) between sequences, encoding for EGFP and the capsid protein were made to increase the overall molecular mass of the recombinant protein [13]. This insertion had no effect on the nuclear transport of WT or ∆NES capsid proteins (Figure 3B). As observed with T1∆NES, T2∆NES accumulated in nucleolus-like structures in the nucleus (Figure 3B). As the product of T2∆NES, fusion protein EGFP-LucFF-T2∆NES, has a molecular mass of 99 kDa it is not expected to passively diffuse into the nucleus. Furthermore, markedly less T3 (107 kDa) was detected in the nucleus when compared to WT (+/−LucFF) protein (Figure 3B). These results indicate that EGFP-LucFF-WT (118 kDa) but not EGFP-LucFF-T3 is actively transported to the nucleus. Taken together, these results suggest that CHIKV capsid protein is actively transported to the nucleus and that amino acids upstream of Q83 are required for this. The precise location of the nuclear localization signal of CHIKV capsid and its functional analysis are the topic of ongoing study.

## 4. Discussion

As positive-strand RNA viruses, alphaviruses replicate within the cytoplasm of host cells. Despite this, several alphaviruses target a number of viral proteins to the nucleus. The rationale for this is not immediately obvious, however, previous studies have highlighted host cell shut-off as one facet of capsid protein nuclear localization in encephalitic alphaviruses [7]. Compartmentalization of viral proteins within subcellular structures of the host cell is therefore an important mechanism used by alphaviruses to augment protein function and optimize infectivity, particularly for a protein with multiple roles during infection such as CHIKV capsid protein.

CHIKV capsid protein localizes to the cytoplasm and the nucleus in over expression studies. Our results outline a minimal NES, with homology to the NES of VEEV capsid protein, consisting of hydrophobic amino acid residues in the N terminal of the capsid protein. The identified NES is recognized as a classical, or prototypical, NES as it conforms to the traditional consensus Φ-X_2–3_-Φ-X_2–3_-Φ-X-Φ where Φ represents leucine, isoleucine, phenylalanine, valine, or methionine and X can be any amino acid. This NES differs from the one previously reported in CHIKV capsid protein, which describes a non-classical sequence towards the centre of the protein [8]. It is therefore possible that CHIKV capsid contains two NESs that must both be intact for functional export of capsid from the nucleus, stressing the importance of capsid protein nuclear shuttling in CHIKV replication. Our results further delineate the nuclear localization signal (NLS) of CHIKV capsid to the N terminal 83 amino acids, which comprises a previously reported NLS between amino acids 60 and 100 [8].

In accordance with previous studies, export of CHIKV capsid protein was CRM1 dependent, with capsid unable to exit the nucleus upon leptomycin B treatment. The active removal of capsid from the nucleus via a CRM1 mediated NES suggests that, in infected cells, capsid is actively exported from the nucleus and that this may be common to all alphavirus capsid proteins encoding this motif. Also, luciferase fusion constructs of CHIKV capsid, which would be excluded from the nucleus by passive diffusion due to their size, were observed in the nucleus confirming that capsid is actively transported to the nucleus. Given that capsid protein is observed predominantly in the cytoplasm of WT CHIKV infected cells, and that in CHIKV ΔNES infected cells capsid protein was observed in the nucleus, our results confirm that capsid actively and rapidly traverses the nucleus during infection.

Interestingly, in this study, mutation of CHIKV capsid protein NES blocked host cell nuclear import. Further studies will be required to understand the block to nuclear import caused when the ∆NES mutation is introduced into CHIKV capsid. Previous studies suggest the formation of a complex containing VEEV capsid is able to accumulate in the nuclear pore complex blocking nuclear import [7]. Furthermore, the relative position and/or sequence between VEEV capsid NES and NLS was important in blocking the nuclear pore complex. Given that we have mapped a NLS in the N terminal 83 amino acids of CHIKV capsid it is possible that mutating the NES of CHIKV capsid permits the formation of a structure capable of blocking nuclear import, similar to that seen with WT VEEV capsid protein. Additionally, in CHIKV ∆NES infected cells, a high proportion of capsid protein was observed at the nuclear periphery indicative of an association with the nuclear pores, as seen with WT VEEV capsid protein [5]. With an abundance of capsid protein found in the cytoplasm during infection, it also remains unclear whether all or just a proportion of capsid protein is able to traffic through the nucleus. The block to nuclear import may also reflect the fact that, in this replicon system, CHIKV capsid is expressed together with CHIKV nsP2, a protein shown to traffic to the nucleus in arthritogenic alphaviruses but that inefficiently translocates to the nucleus in encephalitic alphaviruses [6,14,15]. Although the localization of nsP2 remained unchanged in CHIKV ∆NES infected cells, a block to nuclear import caused by CHIKV ∆NES capsid may represent an important yet undefined role for nsP2 in the trafficking of proteins, including capsid, through the nuclear membrane. The nuclear trafficking of alphaviral proteins warrants further investigation.
